# *Streptococcus pneumoniae* and Influenza A Virus Co-Infection Induces Altered Polyubiquitination in A549 Cells

**DOI:** 10.3389/fcimb.2022.817532

**Published:** 2022-02-24

**Authors:** Thomas Sura, Vanessa Gering, Clemens Cammann, Sven Hammerschmidt, Sandra Maaß, Ulrike Seifert, Dörte Becher

**Affiliations:** ^1^ Department of Microbial Proteomics, Institute of Microbiology, University of Greifswald, Greifswald, Germany; ^2^ Friedrich Loeffler-Institute of Medical Microbiology-Virology, University Medicine Greifswald, Greifswald, Germany; ^3^ Department of Molecular Genetics and Infection Biology, Interfaculty Institute for Genetics and Functional Genomics, University of Greifswald, Greifswald, Germany

**Keywords:** co-infection, ubiquitin, influenza A virus, *Streptococcus pneumoniae* D39, A549

## Abstract

Epithelial cells are an important line of defense within the lung. Disruption of the epithelial barrier by pathogens enables the systemic dissemination of bacteria or viruses within the host leading to severe diseases with fatal outcomes. Thus, the lung epithelium can be damaged by seasonal and pandemic influenza A viruses. Influenza A virus infection induced dysregulation of the immune system is beneficial for the dissemination of bacteria to the lower respiratory tract, causing bacterial and viral co-infection. Host cells regulate protein homeostasis and the response to different perturbances, for instance provoked by infections, by post translational modification of proteins. Aside from protein phosphorylation, ubiquitination of proteins is an essential regulatory tool in virtually every cellular process such as protein homeostasis, host immune response, cell morphology, and in clearing of cytosolic pathogens. Here, we analyzed the proteome and ubiquitinome of A549 alveolar lung epithelial cells in response to infection by either *Streptococcus pneumoniae* D39Δ*cps* or influenza A virus H1N1 as well as bacterial and viral co-infection. Pneumococcal infection induced alterations in the ubiquitination of proteins involved in the organization of the actin cytoskeleton and Rho GTPases, but had minor effects on the abundance of host proteins. H1N1 infection results in an anti-viral state of A549 cells. Finally, co-infection resembled the imprints of both infecting pathogens with a minor increase in the observed alterations in protein and ubiquitination abundance.

## Introduction

Respiratory tract infections are among the most prevalent causes of death worldwide (Roth et al., 2018). These infections are mainly caused by influenza A virus (IAV), *Streptococcus pneumoniae* and other pathogens (Aliberti and Kaye, 2013; Troeger et al., 2019). Infections with IAV can cause dissemination of bacteria to the lower respiratory tract allowing viral and bacterial co-infection (Ruuskanen and Järvinen, 2014; Self et al., 2017). Thus, viral and bacterial co-infection is related to increased mortality compared to single infections (Palacios et al., 2009; Hammerschmidt, 2016; Cawcutt and Kalil, 2017). Recently, it has been shown that co-infection occurs in seasonal and pandemic IAV infection in more than 30% of all cases (Brundage, 2006; Morens et al., 2008). The Gram-positive bacterium *S. pneumoniae* is mainly associated as secondary infection in IAV infected patients (Palacios et al., 2009). Influenza A virus infection can cause disruption of the epithelial barrier and impaired immune response causing bacterial spreading and reduced bacterial killing (Siemens et al., 2017). In the lung, epithelial cells act as an important line of defense and are involved in pathogen sensing and initiation of the host immune response (Vareille et al., 2011; Hiemstra et al., 2015). Among others, posttranslational modifications of proteins, like protein phosphorylation and ubiquitination, regulate processes within the host cell. Proteins can be modified with various types of ubiquitination like monoubiquitination and polyubiquitination. Ubiquitin, a protein of 76 amino acids, is covalently linked to the ε-amine group of lysine residues in target proteins and thus, regulates their stability, localization or interaction properties. The function of ubiquitination depends on the length and linkage type of the ubiquitin chain. Furthermore, ubiquitin is involved in the regulation of immunity and clearance of intracellular pathogens (Bhat and Greer, 2011; Zinngrebe et al., 2014; Hu and Sun, 2016). It was demonstrated previously that bacterial and viral pathogens are able to manipulate the ubiquitin proteasome system (UPS) to evade the host defense. Moreover, host ubiquitin is misused by influenza A viruses to facilitate particle uncoating and transport within the host cell (Rudnicka and Yamauchi, 2016; Yamauchi, 2020). Here, we infected type 2 lung epithelial cells (A549) with a pandemic IAV, with *S. pneumoniae* D39Δ*cps*, and conducted viral and bacterial co-infection to elaborate the effects of these infections on protein abundance and on protein ubiquitination. We enriched for K48 and K63 polyubiquitinated proteins, because these ubiquitin chains are the most dominant within the lung tissue, and applied label free LC-MS/MS based quantification. The results of this study contribute to the understanding of the ubiquitin mediated regulatory processes during viral and bacterial co-infection.

## Materials and Methods

### Bacterial and Viral Strains

*Streptococcus pneumoniae* D39Δ*cps*, a non-encapsulated mutant of D39 was described earlier (Saleh et al., 2013).

The pandemic influenza A/H1N1 virus [A/Germany-BY/74/2009(H1N1)] was kindly provided by the Institute of Immunology (FLI, Federal Research Institute for Animal Health) (Greifswald-Insel Riems, Germany).

### Cell Culture and Infections

A549 alveolar type 2 lung epithelial cells (ATCC CCL-185) were cultured in T75 flasks in RPMI medium (Invitrogen) supplemented with 10% fetal calf serum (FCS) at 37°C in 5% CO_2_ atmosphere. For infection experiments, cells were seeded into T75 flasks and grown to 80-90% confluence. 2 h before all infections the medium of A549 cells was exchanged by RPMI without FCS.

Bacteria were grown at 37°C in Todd Hewitt broth supplemented with 0.5% yeast extract (THY) to mid-exponential phase (OD_600_ of 0.35-0.40). Pneumococci were washed and resuspended in PBS prior to infection. A549 cells were infected with *S. pneumoniae* D39Δ*cps* at a MOI of 15 and were harvested 6 h post infection.

For viral infections, A549 cells were infected at a MOI of 5. After 2 h the medium was replaced by RPMI supplemented with 10% FCS and cells were harvested 24 hours post infection. Viral replication was confirmed *via* qRT-PCR (**Supplementary Figure S1**).

For co-infections, A549 cells were infected with IAV followed by bacterial infection. Cells were infected with IAV at a MOI of 5. After 2 h the medium was replaced by RPMI supplemented with 10% FCS and infected cells were cultured for additional 20 h at 37°C in 5% CO_2_ atmosphere. Then, 2 h before bacterial infection, media was changed to RPMI without FCS. Hereafter, A549 cells were infected with *S. pneumoniae* D39Δ*cps* (MOI 15) for 6 h.

At the end of the infection period cells were washed with PBS and cells were detached using trypsin and counted. Cell counts were determined by light microscopic quantification of cell viability. Cells were stained with 0.4% (v/v) trypan blue under serum-free conditions and visually examined.

All experiments were performed in triplicates (n=3).

### Interleukin ELISA

ELISA plates (Human IL-6/IL-8 Max Deluxe Set [BioLegend]) were coated with capture antibody and incubated for 16-18 h at 4°C. Plates were washed (PBS + 0.5% Tween-20). Hereafter, plates were blocked with blocking solution for 1 h with shaking at 200 rpm. Furthermore, standard series were prepared (IL-8: 1000 pg/ml – 15.6 pg/ml, IL-6: 500 pg/ml – 7.8 pg/ml). Non-specific bindings were blocked, blocking solution was removed and plates were washed. Standards and cell culture medium samples were added to the plates and incubated for 2 h with shaking. After the plates were washed, they were incubated with the detection antibody for 1 h. The detection antibody solution was removed and plates were washed. Avidin-HRP solution was added and incubated for 30 min with shaking. Avidin-HRP solution was removed and plates were washed. TMB substrate solution was added and incubated for 15 min in the dark. Stop solution (2 N H_2_SO_4_) was added and absorbance was measured at 450 nm and 570 nm within 15 min.

### Protein Extraction

Protein extraction was performed as described elsewhere (Sura et al., 2021). Briefly, harvested cells were resuspended in 1 ml lysis buffer (50 mM Tris HCl [pH 7.5], 0.15 M NaCl, 1 mM EDTA, 1% NP 40, 10% glycerol, 1x cOmplete Protease Inhibitor Cocktail [Roche], 1 mM PMSF, 10 mM N-ethylmaleimide, 20 µM MG132), sonicated to shear DNA and cleared by centrifugation. Protein concentration of the samples was determined by the BCA assay.

### Selective Enrichment of K48 and K63 Ubiquitinated Proteins

Lysates containing 500 µg of total protein were transferred into new tubes and filled to 300 µl with lysis buffer. To reduce unspecific binding of proteins to magnetic beads, 10 µl of magnetic control beads (LifeSensors) have been added to the samples and were incubated in an overhead shaker for 1 h at 10 rpm and 4°C. Afterwards, supernatants were transferred into new tubes. For the enrichment of K48 ubiquitinated proteins, 40 µl of K48 magnetic TUBE HF (LifeSensors), washed with Tris-buffered saline with Tween20 (TBS-T), were added, followed by an incubation for 3 h at 10 rpm and 4°C. To enrich K63 polyubiquitinated proteins, 700 µl water and K63 Flag-TUBE (LifeSensors) with a final concentration of 50 nM were added to the precleared sample and incubated for 2 h at 10 rpm and 4°C. Subsequently, 8 µl of magnetic anti-Flag beads were added and samples were incubated for additional 2 h. The unbound fraction of both enrichments was removed and the beads were washed three times with 500 µl TBS-T. To elute the enriched proteins, beads were resuspended in 25 µl SDS buffer (5% SDS; 50 mM TEAB; 5 mM TCEP) and incubated at 65°C for 45 min with 300 rpm shaking. Finally, the supernatants were transferred into new tubes.

### Proteolytic Digest

Extracted and enriched ubiquitinated proteins were digested by suspension trapping on micro S Traps (Protifi) as described elsewhere (Sura et al., 2021). Briefly, lysates containing 30 µg protein were transferred into new tubes and disulfide bonds were reduced by adding TCEP. Thiol groups were alkylated by adding iodoacetamide for proteome samples or chloroacetamide for ubiquitinome samples. Proteins were digested by adding 25 µl digestion buffer (50 mM TEAB) containing 1.2 µg trypsin for proteome samples and 600 ng trypsin (Promega) for ubiquitin enriched samples followed by an incubation for 3 h at 47°C. Peptides were subsequently eluted with 40 µl 50 mM TEAB; 0.1% acetic acid; 60% ACN in 0.1% acetic acid. All eluted fractions of a sample were pooled and dried in a vacuum concentrator. Dried peptides were stored at -80°C

### Peptide Fractionation

Peptides generated from crude protein extracts were fractionated by off-line high pH reversed phase chromatography as described elsewhere (Sura et al., 2021).

### LC-MS/MS Analysis

The peptide composition of the generated samples was analyzed by LC-MS/MS with an EASY-nLC 1000 (Thermo Fisher Scientific) coupled to a QExactive mass spectrometer (Thermo Fisher Scientific). Peptides were loaded onto in-house packed fused silica columns of 20 cm length and an inner diameter of 75 µm, filled with ReproSil Pur 120 C18-AQ 1.9 µm (Dr. Maisch). Peptides were subsequently eluted by a non-linear binary gradient of 165 min from 2% to 99% solvent B (0.1% acetic acid in acetonitrile) in solvent A (0.1% acetic acid). Detailed information on the gradient and LC setup can be found in **Supplementary Table S1**. MS/MS data for the proteome and the K48 ubiquitin chain enriched samples were acquired as previously described (Sura et al., 2021). For the K63 ubiquitin chain enriched samples the mass spectrometer was operated in DDA mode. The survey scan was acquired from 300-1650 m/z with a resolution of 70,000 at 200 m/z. The 10 most abundant ions were selected for fragmentation *via* HCD with a normalized collision energy of NCE 27. The AGC target was set to 1E5 with an underfill ratio of 10% and a maximum injection time of 180 ms. MS/MS spectra were acquired in centroid mode with a resolution of 17,500 at 200 m/z. Ions with unassigned charge states as well as charge 1 and higher than 6 were excluded from fragmentation. Dynamic exclusion was set to 30 s and lock mass correction was enabled. Detailed information on the MS/MS acquisition parameters for all datasets are provided in **Supplementary Tables S2** and **S3**.

### Database Search

Datasets of enriched polyubiquitinated and total proteome samples were processed separately. Raw data were searched with MaxQuant (version 1.6.17.0) (Cox and Mann, 2008; Cox et al., 2014) against the UniProt databases for human (July 2019, UP000005640, 20,416 entries), Influenza A virus (A/Germany-BY/74/2009(H1N1)) (December 2017, UP000153067, 10 entries) and for *S. pneumoniae* D39 (September 2020, UP000001452, 1,915 entries). The maximum number of allowed missed cleavages was 2 and precursor mass tolerance was set to 4.5 ppm. Carbamidomethylation (C) was set as a fixed modification, oxidation (M) and acetylation (protein N‐termini) were set as variable modifications for both datasets. GlyGly (K) was set as an additional variable modification for K48 and K63 ubiquitin chain enriched samples. Proteins were identified with at least two unique peptides, and peptide-spectrum match (PSM) and protein false discovery rate (FDR) were set to 0.01. Match between runs was applied and protein abundances were calculated by the MaxLFQ algorithm. A detailed table of applied parameters for database searching can be found in **Supplementary Tables S4** and **S5**.

### Data Analysis

Identified protein groups were analyzed with Perseus (version 1.6.15.0) (Tyanova et al., 2016; Tyanova and Cox, 2018). Only identified by site and reverse hits, as well as potential contaminations were removed. To assess differentially expressed proteins, a two tailed *t* test was applied for proteins with “LFQ intensity” values in three out of three replicates of the compared groups. Proteins were considered as differentially expressed with a *p* value < 0.05 and a fold change > 1.5. Quantification results and summary statistics can be found in **Supplementary Table S6**. Functional annotation enrichment of proteins with significantly changed abundance was performed with the Database for Annotation, Visualization and Integrated Discovery (DAVID v6.8) (Da Huang et al., 2009a; Da Huang et al., 2009b) and Reactome (Jassal et al., 2020). Protein interaction networks were created with STRING (v11.0) (Szklarczyk et al., 2019). Data were visualized using Inkscape (version 0.92.2) and RStudio (version 1.3.1073) with the packages ggplot2 (version 3.3.5), dplyr (version 1.0.6), EnhancedVolcano (version 1.4.0), splitstackshape (version 1.4.8), shadowtext (version 0.0.8) and ggpubr (version 0.4.0). Data variance was assessed by calculation of CVs from proteins with quantitative values in all three replicates (**Supplementary Figure S2**).

## Results

To investigate the effect of bacterial or viral mono-infections and bacto-viral co-infections we infected A549 cells at a MOI that does not kill the majority of the A549 cells within the analyzed time frame. Also, we aimed to explore, whether the co-infection will be more severe to A549 cells than the infections with a single pathogen. Therefore, we analyzed the proteome and ubiquitinome of mono- and co-infected A549 cells.

### Cell Count and Interleukin Secretion

A549 cells were grown to 80%-90% confluence. Hereafter, cells were challenged either with the influenza A virus H1N1, *S. pneumoniae* D39Δ*cps*, or both pathogens in a viral bacterial co-infection. After 24 h of viral infection the A549 cell count significantly increased by more than 25% (**Supplementary Figure S3**). This indicates that IAV propagation does not interfere with A549 proliferation within the observed time frame. In contrast, a 25% reduced cell count was observed after 6 h of *S. pneumoniae* infection. After co-infection the number of cells did not change compared to the uninfected control.

Cell culture supernatants of each infection were used to determine the levels of secreted immune modulating effectors. The average (n=3) level of IL-6 and IL-8 was increased after *S. pneumoniae* infection and bacto-viral co-infection (**Supplementary Figure S4**), but not after single viral infection. In detail, there is a difference in IL-6 and IL-8 levels when comparing the bacterial single infection or the co-infection with the corresponding mock infection. In contrast, there is no difference between the single H1N1 infection and the associated mock infection. Therefore, the H1N1 infection does not lead to an increased interleukin production.

### Differentially Abundant Proteins in Proteome and Ubiquitinome

**Figure 1:**
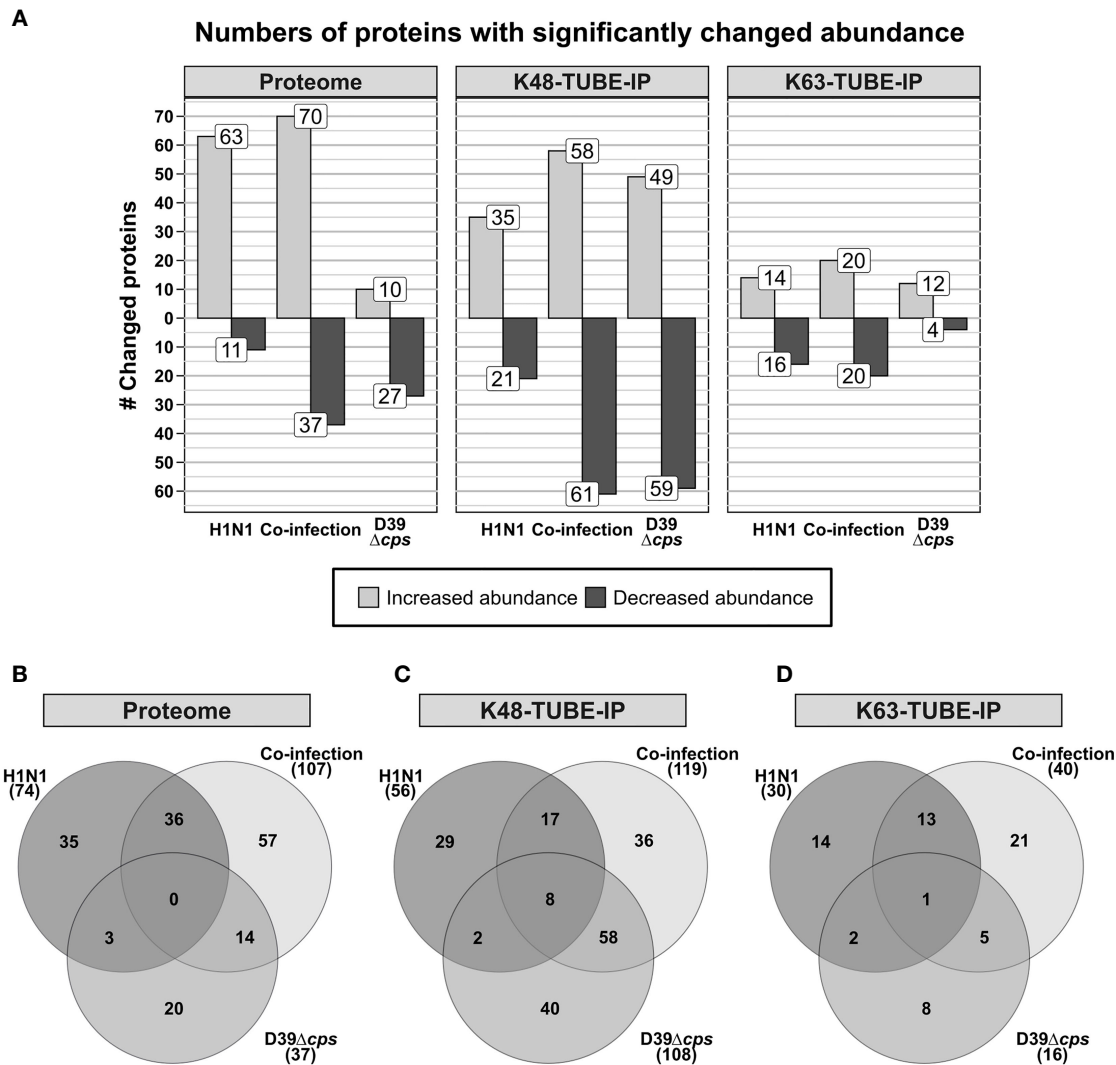
Analysis of differentially abundant proteins and differential abundance in polyubiquitination of A549 cells in response to single and co-infections with influenza A virus H1N1 and *Streptococcus pneumoniae* D39Δ*cps*. **(A)** Number of proteins that have been detected with differential abundance on protein or polyubiquitination level in the different data sets. **(B–D)** Venn diagrams displaying the overlap of proteins detected with differential abundance between the analyzed single infection and co-infection.

For all experiments, the PSM FDR and the protein FDR have been set to 0.01. Proteins were considered as significantly changed in abundance, when they were quantified in all three replicates of the compared groups with a fold change greater than 1.5 and a *p*-value below 0.05. This, in total, resulted in the identification of more than 90,000 peptides, enabling quantification of more than 5,800 proteins for the proteome data set. In the proteome of A549 cells 74, 37 and 107 proteins were found to be significantly changed in abundance upon H1N1 infection, *S. pneumoniae* infection, and co-infection, respectively (**Figure 1A**). H1N1 infection induced the accumulation of various proteins, whereas the *S. pneumoniae* infection mainly led to decreasing protein abundances. Moreover, different sets of proteins were effected by viral or bacterial infection. This is also reflected by the low overlap between these infections (**Figure 1B**).

After enrichment of polyubiquitinated proteins we were able to identify more than 34,000 and 9,800 peptides, from the K48 and K63 enriched samples, respectively. This enabled the quantification of more than 2,800 proteins after K48 enrichment and more than 850 proteins in the K63 enriched data set (**Figure 1A**). In contrast to the proteome and K63 polyubiquitin enriched data sets, *S. pneumoniae* infection induced more changes than H1N1 infection in the K48 polyubiquitin enriched data set. Comparison of the proteins, which were differentially expressed upon viral and bacterial infection, revealed only a limited number of shared proteins between the single infections (**Figures 1A, C, D**). To elaborate whether altered abundance detected for proteins in the polyubiquitin enriched data sets originates from alterations in protein abundance in general, we examined the overlap of proteins that were detected with altered abundance in those data sets (**Supplementary Figure S5**). Whereas upon pneumococcal infection three proteins were detected with differential expression in the proteome and K48 enriched samples, 18 and 15 proteins were differentially expressed in the proteome and K48 enriched data set upon viral and co-infection, respectively (**Supplementary Figure S5**). This indicates that in most cases altered abundance in polyubiquitin enriched samples is independent of variations in the abundance of the respective proteins. Hence, differential abundance in polyubiquitin enriched data sets is caused by changing polyubiquitination incidence rather than by changing protein abundance. Volcano plots and MA plots, comparing the different conditions are shown in **Supplementary Figures S6**–**S8**.

### Functional Analysis of Proteins Differentially Expressed in the Total Proteome Samples

To get detailed insights into the processes driving the cellular response to the infecting pathogens, we used the Database for Annotation, Visualization and Integrated Discovery (DAVID) to perform annotation enrichment on proteins with significantly changed abundance.

**Figure 2:**
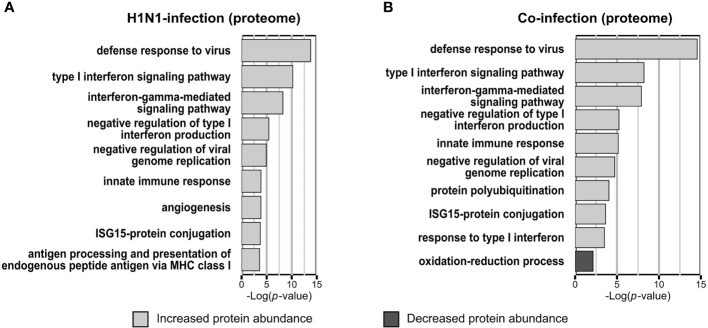
Gene ontology biological process (GOBP) terms enriched from proteins with significantly changed abundance. Categorical annotation enrichment (*p*-value < 0.01) of GOBP terms from proteins with significantly altered abundance (*p*-value < 0.05; fold change > 1.5) in the proteome. Enrichment analysis of A549 cells infected with Influenza A virus **(A)** or co-infected with *Streptococcus pneumoniae* D39Δ*cps* **(B)** [n=3]. **(A, B)** Displayed are the top hits of the enriched GOBP terms from proteins with increased and decreased abundance, ranked by the enrichment *p*-value.

Infecting A549 cells with H1N1 significantly altered the abundance of 74 proteins within the proteome samples. The abundance of 63 proteins was increased and decreased for eleven proteins. Functional annotation enrichment revealed that *type I interferon*, *ISG* and other terms, which represent a response to viruses, were mainly enriched within proteins of increased abundance (**Figure 2A**). No terms have been enriched from proteins with reduced abundance (**Figure 2A**).

In the proteome of *S. pneumoniae* infected A549 cells only 37 proteins were detected with significantly altered abundance, among them we detected ten with increased abundance and 27 with decreased abundance. Here, the terms *oxidation-reduction process* (*p*-value = 0.00022) and *regulation of translation* (*p*-value = 0.0055) were enriched from proteins with reduced abundance. In contrast to the H1N1 infection, no terms were enriched from proteins with increased abundance.

Viral and bacterial co-infections of A549 cells mainly affected Gene Ontology Biological Process-terms (GOBP-terms) similar to those enriched in single infections. Proteins with increased abundance were annotated for GOBP terms *defense to virus*, *type I interferon signaling pathway* and *ISG15-protein conjugation*. The term *oxidation-reduction process* was enriched from proteins with decreased abundance (**Figure 2B**). In general, there is a reasonable quantity of proteins detected with the same regulation between the single infection and the co-infection (**Figure 1B**). Additionally, several proteins were quantified in the H1N1 and co-infection, but were missing in uninfected and *S. pneumoniae* infected samples. These proteins are MX1, MX2, IFI44, IFIT1, IFIT2, IFIT3 and IFIH1, which are all involved in *type I interferon signaling* and viral defense.

Furthermore, 17 proteins that are included in the integrated annotations for Ubiquitin and Ubiquitin-like Conjugation Database (iUUCD 2.0) (Zhou et al., 2018) and 2 proteins that are catalytically active subunits of the immunoproteasome as well as the proteasome activator subunit beta (PA28beta) were detected with altered protein abundance upon infection (**Table 1**).

**Table 1:** List of proteins that were detected with differential abundance upon infection and are listed in the iUUCD 2.0 database or related to the immunoproteasome and proteasome regulation (highlighted with an asterisk).

Protein	iUUCD 2.0		fold change		
	family	ID	H1N1	Co-infection	D39
TMF1	E3 adaptor	IUUC-Hsa-046889	1.09	**-1.81**	-1.33
CIAO1	E3 adaptor	IUUC-Hsa-046528	–	**-1.79**	-1.43
CORO7	E3 adaptor	IUUC-Hsa-045847	1.43	**1.93**	**1.94**
MNAT1	E3	IUUC-Hsa-045876	**1.58**	1.34	1.19
RBCK1	E3	IUUC-Hsa-045738	**1.66**	1.77	-1.45
IRF2BPL	E3	IUUC-Hsa-046942	1.16	1.51	1.13
TRIM56	E3	IUUC-Hsa-046238	**1.59**	**1.55**	1.08
TRIM25	E3	IUUC-Hsa-045828	**1.67**	**1.58**	1.03
RNF213	E3	IUUC-Hsa-046140	**1.64**	**1.99**	-1.10
RNF121	E3	IUUC-Hsa-046468	–	1.53	**1.57**
PML	E3	IUUC-Hsa-045861	**3.57**	**4.19**	1.08
TRIM21	E3	IUUC-Hsa-046583	**5.17**	**4.62**	-1.44
DTX3L	E3	IUUC-Hsa-045921	**19.49**	**18.17**	-1.10
HERC5	E3	IUUC-Hsa-046342	**12.21**	**28.65**	–
UBE2T	E2	IUUC-Hsa-046513	1.31	-1.41	**-1.54**
UBE2E1	E2	IUUC-Hsa-046576	1.04	-1.95	**-1.53**
UBE2L6	E2	IUUC-Hsa-045943	**5.23**	**7.72**	1.33
PSME2*	–	–	1.27	**1.53**	1.15
PSMB9*	–	–	1.67	**1.95**	-1.11
PSMB8*	–	–	**1.76**	**2.34**	-1.02

The fold change upon each infection is given and highlighted in bold if the detected change is statistically significant (students *t*-test; *p*-value < 0.05; fold change > 1.5; n=3).

### Functional Analysis of Differentially Abundant Proteins in K48 Enriched Samples

As ubiquitination is involved in the regulation of nearly every cellular process, we selectively enriched for K48 and K63 polyubiquitinated proteins. It was demonstrated that K48 and K63 polyubiquitin chains are the most abundant chain types in murine primary macrophages and in murine lung tissue (Heunis et al., 2020). K48 ubiquitination has a variety of functions in the eukaryotic cell. Still, the primary function is to mark proteins for proteasomal degradation.

After 24 h of H1N1 infection, there were fewer changes in protein abundance detected in K48 enriched samples compared to total proteome samples. GOBP terms that were most significantly overrepresented from proteins with increased abundance in the K48 enriched samples were *defense response to virus*, *type I interferon signaling pathway* and *negative regulation of viral genome replication*, while *RNA splicing* was most significantly overrepresented from proteins with decreased abundance.

Upon *S. pneumoniae* infection we detected altered abundance of 108 putatively K48 polyubiquitinated proteins. Fifty-nine proteins were quantified with decreased abundance and 49 with increased abundance after infection. Only three out of 37 proteins detected with differential expression in the proteome samples were detected with altered abundance in the K48 polyubiquitin enrichment. Proteins with decreased abundance in K48 polyubiquitination mostly affected processes involved in *mRNA splicing* and *rRNA processing*. *Arp2/3 complex mediated actin nucleation* and other processes related to actin filament organization were highly overrepresented by proteins with increased K48 polyubiquitination. In addition to actin filament related processes, *FC-gamma receptor signaling pathway involved in phagocytosis* was enriched from proteins with increased K48 polyubiquitination, as well.

**Figure 3:**
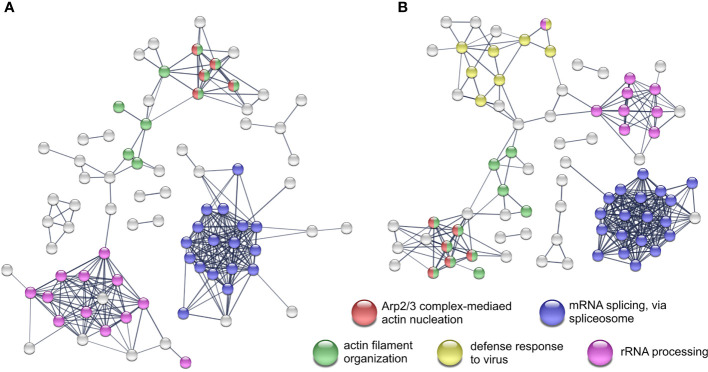
STRING network analyses and the enriched Reactome pathways from K48 polyubiquitinated proteins. Network from proteins with differential expression after *Streptococcus pneumoniae* D39Δ*cps* single-infection **(A)** and co-infection with IAV H1N1 **(B)** All experiments were performed in triplicates (n=3). Networks with protein names are included as **Supplementary Figures S10** and **S11**.

In the viral and bacterial co-infection the pattern of proteins with significantly changed abundance after K48 enrichment is similar to both of the single infections. Moreover, the co-infection shares ~50% and ~15% of differentially expressed proteins with the bacterial and the viral infection, respectively (**Figure 1C**). Proteins that were shared between the single infections change their abundance into the same direction. Although the STRING network, which was generated from the co-infection (**Figure 3B**) data, has a high similarity to the network generated from the bacterial infection (**Figure 3A**), it also contains a cluster of proteins that has been detected upon viral infection (*response to virus*) (**Figure 3B**). Furthermore, eight proteins were found to be changed significantly in all infections and three of them are related to *RNA splicing* (**Figure 1C**). Ten proteins were only quantified in the viral and in co-infection samples. These proteins affect the processes of *response to virus*, *type I interferon signaling pathway* and *interferon-gamma-mediated signaling pathway*.

### Functional Analysis of Differentially Abundant Proteins in K63 Enriched Samples

In contrast to K48 polyubiquitination, K63 polyubiquitin chains are thought to mediate non-proteasomal signals for endocytosis and immune responses. The K63 enriched data set displays the lowest number of quantified proteins within the three data sets obtained in this work. Accordingly, the K63 data set reveals fewer proteins detected with altered abundance. H1N1 infection induced variations in the K63 polyubiquitination level of 30 proteins. Proteins with reduced K63 ubiquitination affect *mRNA splicing* processes and proteins with increased K63 ubiquitination affect *redox* processes. Changes induced by the *S. pneumoniae* infection do not affect multiple pathways, only *negative regulation of mitochondrial outer membrane permeabilization involved in apoptotic signaling pathway* is affected by proteins with increased K63 polyubiquitination. Co-infection most significantly affected processes involved in *mRNA processing* from proteins with decreased polyubiquitination. Proteins with increased polyubiquitination affected *redox* processes and *response to ER stress*.

## Discussion

In the work presented, we used *in vitro* cell culture based infection experiments to examine potential synergistic effects of the infecting pathogens in viral and bacterial co-infections on human lung epithelial cells. Therefore, we analyzed viral and bacterial mono-infections, along with bacto-viral co-infections of A549 cells, focusing on the proteome, as well as the selective enrichment of polyubiquitinated proteins. Infecting A549 cells with H1N1 for 24 h induced a pronounced immune response and viral defense of the host. The additional bacterial infection was performed at MOI 15 to be able to elaborate a potential synergisms of the pathogens.

### Response to IAV H1N1 Infection

Influenza H1N1 infection induced a strong viral defense response of the A549 cells. This was established by the induction of the JAK-STAT cascade, type I interferon signaling, interferon γ signaling and induction of the ISG15 protein conjugation. Induction of the type I interferon production mediated by RIG-I and MAVS, which leads to the induction of a wide array of genes, can be assumed from increased protein abundance of interferon stimulated genes (Schoggins et al., 2011; Zhang et al., 2020). In addition, we detected a 25 fold increase in the abundance of DDX60 which was shown to be required for RIG-I mediated type I interferon expression after viral infection (Miyashita et al., 2011). From the proteins with higher abundance after viral infection, nine show E3 ligase activity and one protein is described as an E2 enzyme (Zhou et al., 2018) (**Table 1**). Among these proteins are HERC5 and TRIM25, both acting as E3 ligating enzymes for ISG15 (Wong et al., 2006; Mathieu et al., 2021), which increased twelve and five times in abundance in our experiments, respectively. UBE2L6, the ISG15 E2 transferring enzyme, was detected to be fivefold more abundant after viral infection. These results are in concordance with previously published data where an up-regulation of UPS genes in response to Interferon-stimulation was detected (Seifert et al., 2010). This, together with the increased abundance of the transcription factor IRF9, which induces the transcription of interferon stimulated genes (ISG) when forming a complex with STAT1 and STAT2, underpins the interferon mediated activation of the JAK-STAT pathway. OAS1-3 are degraders of viral RNA (Shim et al., 2017) and were also detected with increased abundance after viral infection. Moreover, increased protein abundance of the ubiquitin E3 ligase DTX3L and the interacting protein PARP9 as well as of PARP10, 12 and 14 was observed in IAV infected A549 cells. These results confirm the findings on increased protein abundances of PARP9 and PARP14 upon viral infection, first observed by Becker and colleagues (Becker et al., 2018). We were able to confirm these results using a different influenza strain, but the same cell line. It is reported by others that the outcome of the IAV infection depends not only on the MOI and the duration of the infection, but also on the cell line, as well as the employed viral stain (Becker et al., 2018; Wu et al., 2019). Influenza virus infection predispose the host cell to secondary bacterial infections, which was shown from mice and *in vitro* experiments (Sharma-Chawla et al., 2016; Bai et al., 2021). Bai and coworkers demonstrated that viral infection induced an increase in the abundance of PPIA, which interacts and mediates protection from proteasomal degradation of PTK2. Ultimately, this results in increased integrin α5 expression and actin rearrangement, which renders the host cell more susceptible for secondary bacterial infection (Bai et al., 2021). In contrast, we have not detected significant changes in the abundance of PPIA and integrin-α5 in the proteome nor in the abundance of PTK2 in the proteome and K48 enriched data set. These differences in the outcome of the viral infection can potentially be caused by several reasons. First, observed differences were caused by the use of different influenza strains. The second reason can be the infection duration and the MOI at which the experiments were conducted. Bai et al. showed that the mRNA level of PPIA reached its maximum at 12 hpi and decreased afterwards (Bai et al., 2021). The use of a higher MOI may result in a shifted maximal mRNA level and CypA expression. The reason causing the observed differences remains unclear. We can exclude the cell line as a cause, as we used A549, too. In addition, Becker et al. have not observed changes in the protein abundance of CypA or ITGA5 after IAV infection of A549 cells, as well (Becker et al., 2018).

### Response to *Streptococcus pneumoniae* Infection

The pneumococcal infection induced differential abundance of 37 proteins. Furthermore, we detected 108 and 16 proteins with significantly altered abundance in K48 and K63 polyubiquitination, respectively.

The protein that showed the most prominent increase in abundance (about four fold) was PTGS2 or COX-2, being an inducible protein involved in the initial step of prostaglandin synthesis. In type II alveolar epithelial cells the induction of the COX-2 expression in response to pneumococci is controlled in a p38 MAPK and NFκB dependent manner (N'Guessan et al., 2006; Szymanski et al., 2012). Bootsma and colleagues also observed an induction of COX-2 by the Δ*cps* mutant of *S. pneumoniae* analyzing the transcriptional response of Detroit 562 pharyngeal epithelial cells to the adherence of different pneumococcal strains (Bootsma et al., 2007). They have shown that *S. pneumoniae* D39 and its isogenic Δ*cps* mutant alter the expression of different gene sets. Infection with the Δ*cps* mutant changed the expression of 156 genes (28 upregulated and 128 downregulated) (Bootsma et al., 2007). Given the fact that Detroit 562 cells are of pharyngeal origin and A549 cells are lung epithelial cells, the published results cannot be entirely transferred to our study. Nevertheless, Bootsma et al. also observed an increase in IL-6 and IL-8 levels upon *S. pneumoniae* D39Δ*cps* infection (Bootsma et al., 2007) (**Supplementary Figure S4**).

Bacterial pathogens such as *S. pneumoniae* use different strategies like transmigration or transcytosis to cross epithelial or endothelial barriers of the host. Pneumococcal uptake by A549 cells is low, however, A549 cells show increased uptake of *S. pneumoniae* D39Δ*cps* compared to the wild type strain (Talbot et al., 1996; Bootsma et al., 2007). Still, it was shown that pneumococcal phosphorylcholin mimics the natural ligand of PAFr initiating bacterial uptake in a β-arrestin dependent manner (Radin et al., 2005; Bertuzzi et al., 2019). Pneumococcal endocytosis is clathrin and caveolae mediated and the vast majority of endocytosed pneumococci is killed by lysosomal fusion of the endosome (Gradstedt et al., 2013). Pneumolysin, a cholesterol-dependent cytolysin expressed on the pneumococcal surface and released by autolysis, disables the acidification of the endosome by the formation of pores in the endosomal membrane (Barnett et al., 2015; Ogawa et al., 2018). The ubiquitin-proteasome system (UPS) is involved in the autophagosomal entrapment and killing of cytosolic pneumococci (Iovino et al., 2014; Ogawa et al., 2018). Additionally, pneumolysin directly interacts with actin and activates small GTPases leading to remodeling of the actin network (Hupp et al., 2013). Rho GTPases act upstream of the Arp2/3 complex, which facilitate actin nucleation and is involved in clathrin-mediated endocytosis and phagocytosis (Sirotkin, 2011; Sumida and Yamada, 2015; Jin et al., 2021). In our study, we observed increased abundance in K48 polyubiquitination of proteins belonging to the Arp2/3 complex and other actin and cytoskeleton organization related GO terms (**Figure 3A**). Moreover, the Reactome analysis revealed that Rho-GTPase signaling is affected by proteins with increased K48 polyubiquitination, as well. Thereby, polyubiquitination bridges the host proteome and ubiquitinome in response to pneumococcal infection. These results are not in contrast to the finding that *S. pneumoniae* D39 effectively overcomes the epithelial barrier by transmigration (Attali et al., 2008). We have not observed any changes in the abundance of junction proteins as it was described by Peter and colleagues (Peter et al., 2017). This could be due to the use of a single cell line and no lung tissue.

In our K48 polyubiquitin analysis we detected reduced polyubiquitination in proteins involved in mRNA splicing and rRNA processing. Proteins involved in mRNA splicing mainly belong to the spliceosomal E complex. Changes in the spliceosome composition may indicate an early apoptotic state of the epithelial cells, linking the spliceosome and apoptosis (Schwerk and Schulze-Osthoff, 2005). It remains unclear what causes these effects upon pneumococcal infection and is a subject of future studies.

In addition to the K48 polyubiquitin analysis, Reactome analysis of proteins with altered abundance in K63 polyubiquitination also indicated involvement of the Rho-GTPase cycle, whereas the functional annotation enrichment analysis of K63 polyubiquitinated proteins showed only *negative regulation of mitochondrial outer membrane permeabilization involved in apoptotic signaling pathway* as an overrepresented GOBP term.

### Viral and Bacterial Co-Infection

Bacterial and viral co-infections are related to increased severity of disease, increased morbidity and mortality (Siemens et al., 2017; Lim et al., 2019). Influenza A virus infection, caused by pandemic or seasonal strains, predispose the host to secondary bacterial infections (Sharma-Chawla et al., 2016; Siemens et al., 2017; LeMessurier et al., 2020). In our experiments we could not observe an increased cytotoxicity of the co-infection compared to bacterial or viral single infection (**Supplementary Figure S3**). Following the co-infection we observed similar IL6 and IL8 level compared to the bacterial single infection (**Supplementary Figure S4**). Unexpectedly, preceding viral infection reduced pneumococcal adherence to A549 epithelial cells in our experimental setup (**Supplementary Figure S9**). Still, viral and bacterial co-infection resulted in the highest number of proteins with significantly altered abundance on protein and polyubiquitination level. Interestingly, we did not detect over representation of pathways that have not been identified upon one of the two single infections. For all obtained data sets the pathways affected by co-infection resembles both of the single infections. This is in congruence with a previous study where we reported additive effects of IAV H1N1 and *Streptococcus pyogenes* co-infection of 16HBE cells (Sura et al., 2021). Nevertheless, here we have detected enhanced alteration of protein abundance and polyubiquitination incidence upon co-infection. This effect on protein abundance was also noticed for PSMB8, PSMB9 and for PSME2 (**Table 1**). These proteins are immunoproteasome subunits and proteasome activator PA28beta and are involved in altered generation of MHC I antigenic peptides presented to CD8 T-cells (Keller et al., 2015; McCarthy and Weinberg, 2015). The further increase in protein abundance upon co-infection might be induced by enhanced IFN-γ production caused by the bacterial superinfection as it was reported by Strehlitz and coworkers from mice experiments (Strehlitz et al., 2018). Upon co-infection higher numbers of proteins with significantly altered abundance were detected in all data sets when compared to the single infections. Still, these observations are not reflected by an increased cytotoxicity of the viral bacterial co-infection.

In the presented study we used *S. pneumoniae* D39Δ*cps*, an isogenic mutant that lacks the capsular polysaccharides. Thus, the outcome of the study and the observed alterations in the proteome and the ubiquitinome were potentially influenced by the absence of capsular polysaccharides. However, the interaction between A549 cells and the pneumococci is mainly based on bacterial adherence to the cell surface (Agarwal et al., 2010). It has been shown that the intimate contact of pneumococci with host cells is associated with a reduction of CPS (Hammerschmidt et al., 2005). While encapsulated wild-type pneumococci interact only moderately with non-professional host cells under *in vitro* conditions, binding can be enhanced using isogenic non-encapsulated mutants. Thereby, the induced signal transduction cascades can be elucidated and the host responses analyzed. Although this is not related to the pathophysiological conditions under *in vivo* conditions, important discoveries were made in the last decades using this combination of bacteria and host cells.

Importantly, we chose a MOI of only 15 bacteria per host cell to reduce the level of host cell damage caused by pneumolysin and hydrogen peroxide to a minimum in the bacterial single infections and co-infections. In *in vivo* studies higher bacterial infection doses are applied to study the effects in experimental acute pneumonia or septicemia infection models.

In *in vivo* experiments, co-infection appears to be adverse for the host, caused by the IAV induced expression of type I interferons suppressing the bacterial clearance by disturbed recruitment of immune cells (LeMessurier et al., 2020; Park et al., 2021). On the other hand, type I interferons increase the expression of tight junction proteins and decrease the expression of PAFr, reducing pneumococcal uptake and hindering transmigration (LeMessurier et al., 2013). The *in vitro* study presented here is based on A549 cells grown as a monolayer. Due to the lack of immune cells and their interaction with the epithelial barrier in our setup, this adverse effect was probably not observed. Furthermore, A549 cells grown as a monolayer do not form tight junctions (Carterson et al., 2005). The scarcity of tight junctions might explain the fact that we did not observe changes in the expression of tight junction proteins, whether induced by type I interferons, or by pneumococcal infection, as it was observed in lung tissue (LeMessurier et al., 2013; Peter et al., 2017).

It can be concluded that IAV and *S. pneumoniae* D39Δ*cps* co-infection of monolayer grown A549 cells shows additive, but in the current setup no observable synergistic effects. It would be interesting to investigate whether this changes in polarized, 3D grown A549 cells and to determine the impact of the pneumococcal virulence factor pneumolysin.

## Data Availability Statement

The mass spectrometry proteomics data have been deposited to the ProteomeXchange Consortium (http://proteomecentral.proteomexchange.org) via the PRIDE (Perez-Riverol et al., 2019) partner repository with the dataset identifier PXD028465.

## Author Contributions

Conceptualization: TS, US, and DB. Formal analysis: TS. Funding acquisition: SH, US and DB. Methodology: TS and VG. Data analysis: TS. Writing—first draft: TS. Writing—review and editing: TS, VG, CC, SM, SH, US, and DB. All authors contributed to the article and approved the submitted version.

## Funding

This research was funded by the Mecklenburg-Pomerania Excellence Initiative (Germany), the European Social Fund (ESF) Grant KoInfekt (ESF/14-BM-A55-0008/16 and ESF/14-BM-A55-0009/16), and the Helmholtz Institute (ZoonFlu).

## Conflict of Interest

The authors declare that the research was conducted in the absence of any commercial or financial relationships that could be construed as a potential conflict of interest.

## Publisher’s Note

All claims expressed in this article are solely those of the authors and do not necessarily represent those of their affiliated organizations, or those of the publisher, the editors and the reviewers. Any product that may be evaluated in this article, or claim that may be made by its manufacturer, is not guaranteed or endorsed by the publisher.
