# Supplementary material for: Streptococcus pneumoniae and Influenza A Virus Co-Infection Induces Altered Polyubiquitination in A549 Cells
Source: Front Cell Infect Microbiol. 2022 Feb 24;12:817532. doi: 10.3389/fcimb.2022.817532 (PMC8908964; doi:10.3389/fcimb.2022.817532)
Supplement: Supplementary file 1 [file DataSheet_1.pdf]

Supplementary information

Table of content:

Figure S1: Quantification of viral NP mRNA

Figure S2: Violin plots showing the distribution of the calculated CVs for all data sets

Figure S3: Cell counts obtained at the final time point of infection

Figure S4: Determination of secreted interleukin IL6 and IL8 levels upon infection

Figure S5: Overlap of differentially expressed proteins detected in the proteome, K48 and K63 polyubiquitin enriched data sets

Figure S6: Results of the differential expression analysis comparing the uninfected control with the respective infection of proteome samples displayed as volcano plots and as MA-plots

Figure S7: Results of the differential expression analysis comparing the uninfected control with the respective infection of K48 polyubiquitin enriched samples displayed as volcano plots and as MA-plots

Figure S8: Results of the differential expression analysis comparing the uninfected control with the respective infection of K63 polyubiquitin enriched samples displayed as volcano plots and as MA-plots

Figure S9: Pneumococcal adherence to host cells

Figure S10: STRING network from K48 polyubiquitin enriched proteins with differential expression after *Streptococcus pneumoniae* D39Δ*cps* single-infection

Figure S11: STRING network from K48 polyubiquitin enriched proteins with differential expression after influenza A virus H1N1 and *Streptococcus pneumoniae* D39Δ*cps* co-infection

Table S1: LC parameters

Table S2: MS/MS parameters for data acquisition for proteome and K48 enriched samples

Table S3: MS/MS parameters for data acquisition of K63 enriched samples

Table S4: MaxQuant parameters for protein identification and quantification to analyze the proteome

Table S5: MaxQuant parameters for protein identification and quantification to analyze K48 and K63 enriched samples

Table S6: Filtered MaxQuant output and summary statistics for all data sets (provided as a separate file)

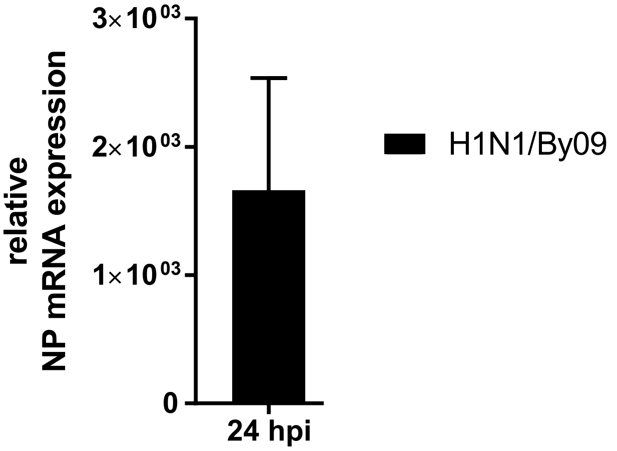

Figure S1: Relative amount of viral NP mRNA quantified by qPCR. The change in viral NP mRNA amount quantified 24 hours post infection (hpi) is related to the 2 hpi NP mRNA expression level. The experiment was conducted in two biological replicates with two technical replicates, each. A549 cells were infected at a MOI of 5 to check for viral replication of the selected influenza A virus strain (A/Germany-BY/74/2009(H1N1)). After 2 h of infection the medium was replaced by RPMI supplemented with 10% FCS, and cells were harvested 24 hpi. Viral replication was confirmed via qPCR. In the chosen experimental setup, infection of A549 cells with the selected virus happens within the first 2 hours of infection which are conducted in FCS free condtion.

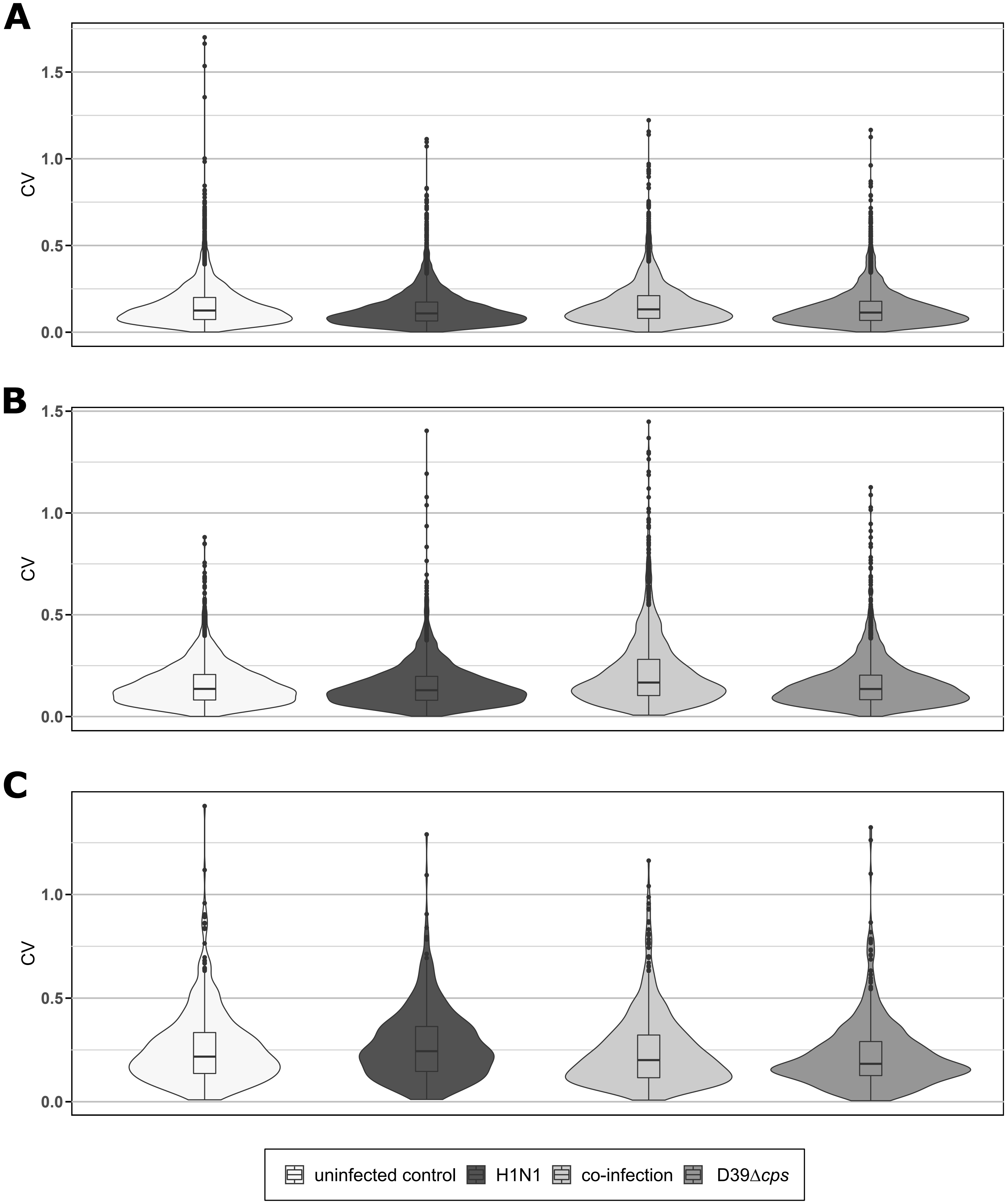

Figure 2: Violine plots showing the distrebution of the calculated CVs for the proteome samples (**A**), for the K48 polyubiquitin enriched samples (**B**) and for the K63 polyubiquitin enriched samples (**C**). CVs were calculted from proteins with quantitative values in all three biological replicates, as only those proteins were used for statistical analysis of the data sets.

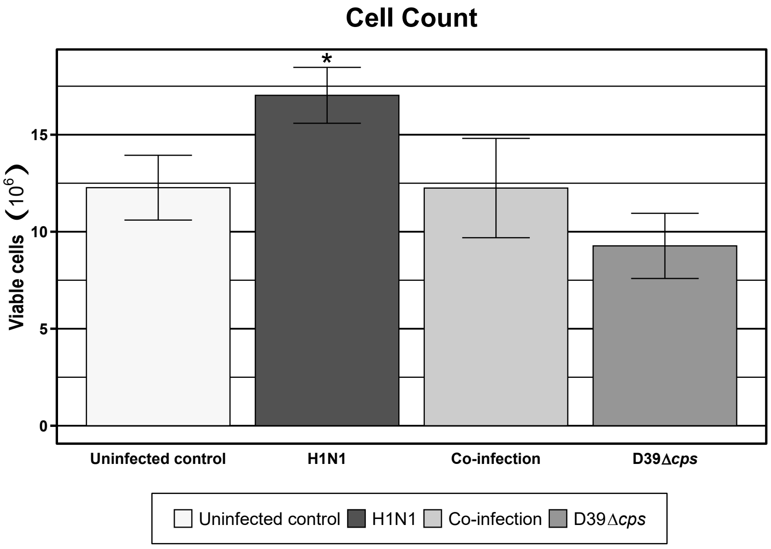

Figure S3: Viable cell counts obtained at the final time point of Infection. A549 cells were infected for 24 h with influenza A virus A/Germany-BY/74/2009 (H1N1) at MOI 5 and 6 h with *Streptococcus pneumoniae* D39Δ*cps* at MOI 15. In co-infection A549 cells were first infected with IAV H1N1 for 24 h followed by 6 h of bacterial infection. Cells were stained with 0.4% trypan blue under serum-free conditions and visually examined. The experiment was performend in triplicates (n=3); * significant alteration in the number of viable cells compared to the uninfected control (*t*-test - *p*-value < 0.05)

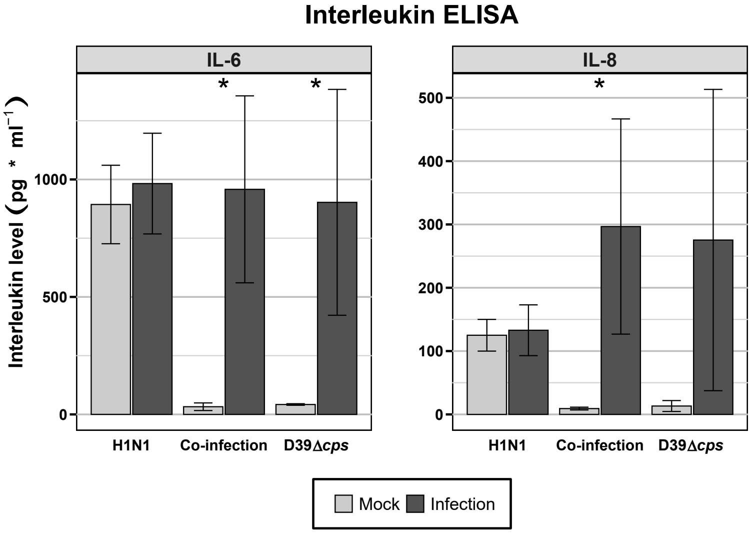

Figure S4: Determination of secreted interleukin levels after single infection with influenza A virus A/Germany-BY/74/2009 (H1N1) or with *Streptococcus pneumoniae* D39Δ*cps* and co-infection. IL-6 and IL-8 levels were determined by ELISA. The experiment was performed in triplicates (n=3); * significant alteration in interleukin level compared to the corresponding mock infection (*t*-test - p-value < 0.05). The mock infection interleukin level corresponding to the virus single infection is elevated due to A549 cell proliferation which results in secretion of IL-6 and IL-8. This is also true for the co-infection setup. However, in the co-infection experiment, A549 cells were washed after H1N1 infection and the medium was replaced with RPMI without FCS. Therefore, elevated IL6 and IL8 level were detected in the mock infections that corresponds to the virus alone infection, but not in the other mock infections.

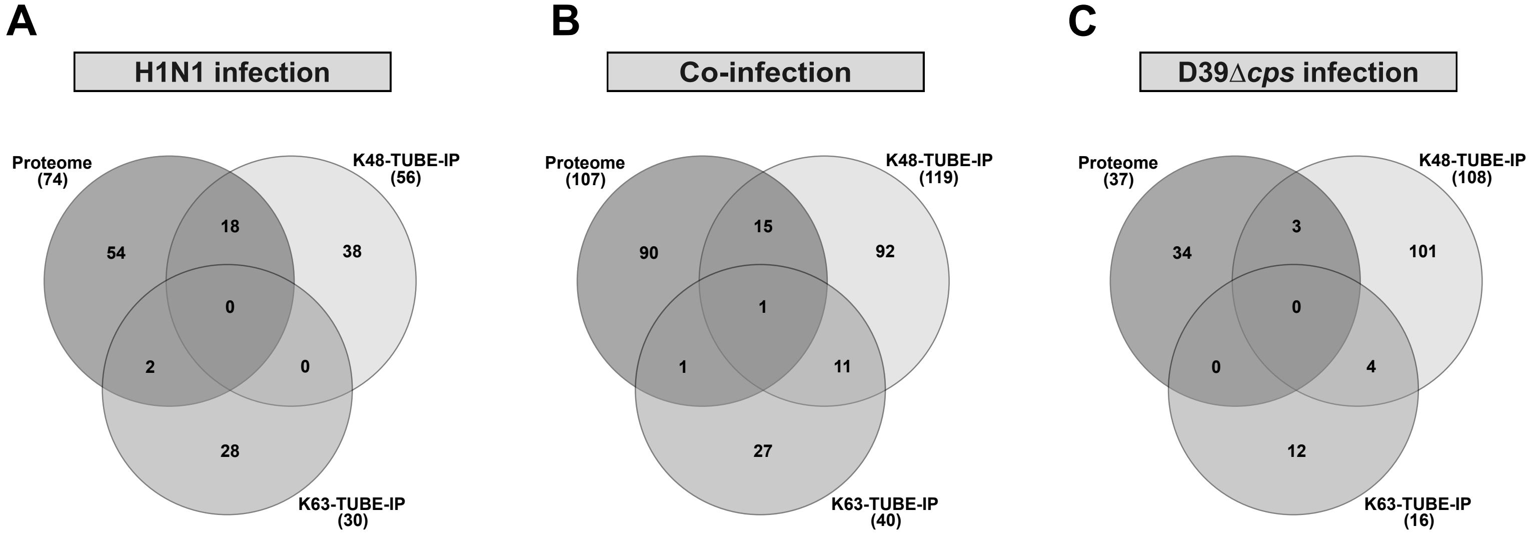

Figure S5: Overlap of differentially expressed proteins (p-value < 0.05; fold change > 1.5) detected in the proteome, K48 and K63 polyubiquitin enriched data sets for IAV H1N1 infection (**A**), *Streptococcus pneumoniae* D39Δ*cps* infection (**B**) and co-infection (**C**).

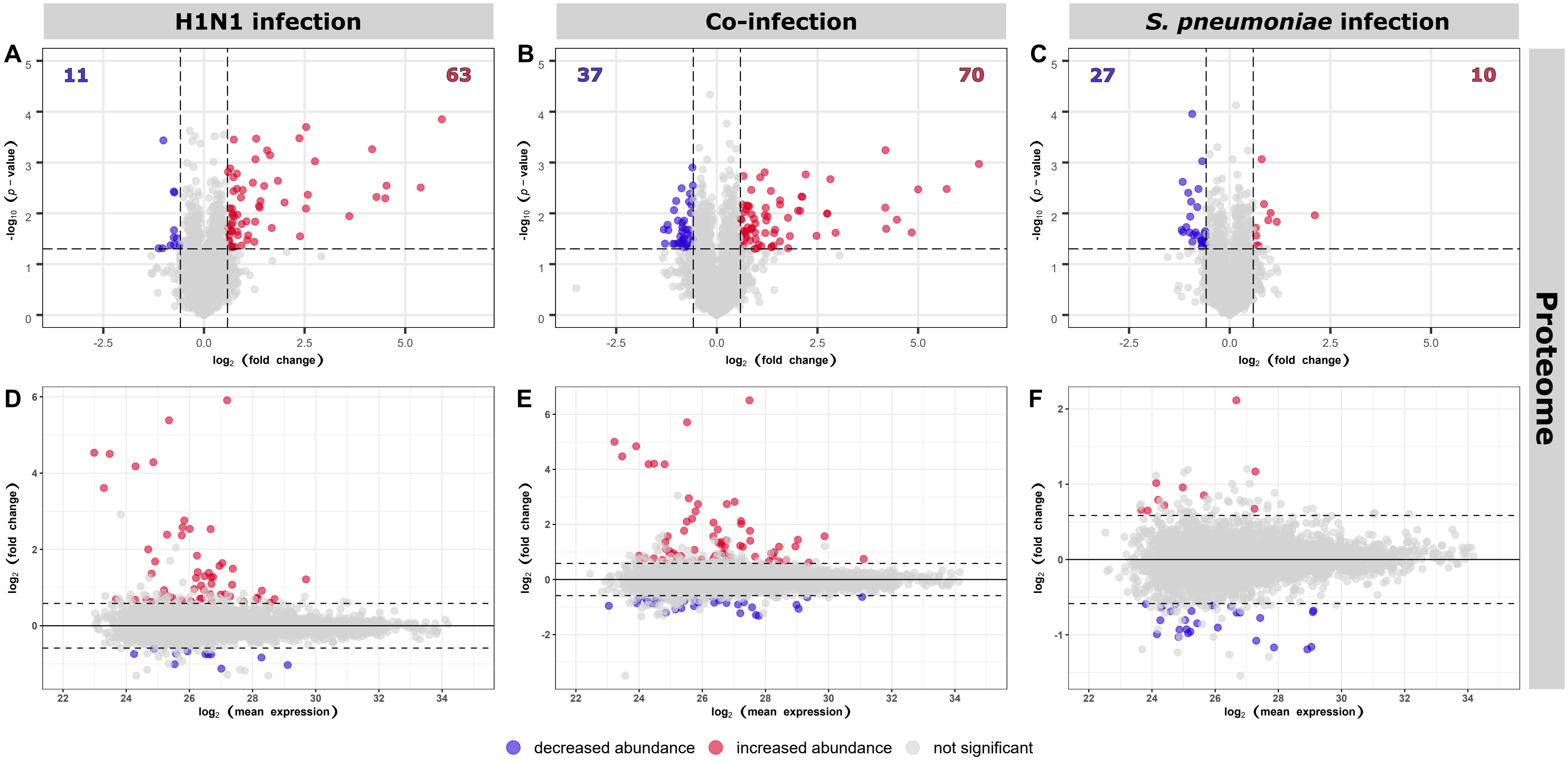

Figure S6: Results of the differential expression analysis comparing the uninfected control with the respective infection (**A** and **D** - H1N1 infection; **B** and **E** - co-infection; **C** and **F** - *S. pneumoniae* D39Δ*cps* infection) of proteome samples displayed as volcano plots (-$\log_{10}$ *p*-value vs. $\log_2$ expression level) (**A, B, C**) and as MA-plots ($\log_2$ expression level vs. $\log_2$ mean expression) (**D, E, F**). Proteins were considered as differentially expressed with a *p*-value < 0.05 and a fold change > 1.5 (students *t*-test). Only proteins with quantitative values in all biological replicates (n=3) of both compared groups were tested.

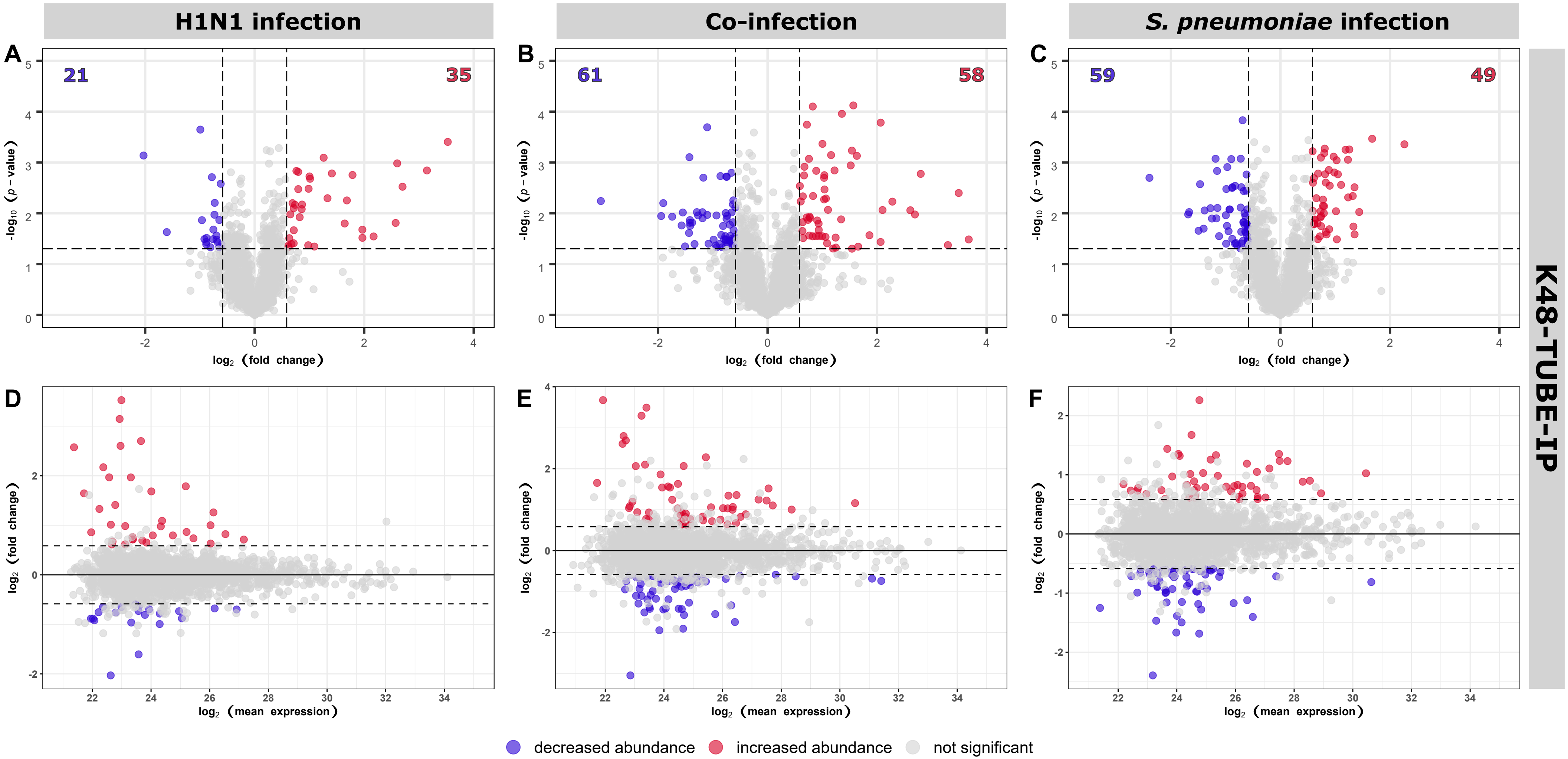

Figure S7: Results of the differential expression analysis comparing the uninfected control with the respective infection (**A** and **D** - H1N1 infection; **B** and **E** - co-infection; **C** and **F** - *S. pneumoniae* D39Δ*cps* infection) of K48 polyubiquitin enriched samples displayed as volcano plots (-$\log_{10}$ *p*-value vs. $\log_2$ expression level) (**A, B, C**) and as MA-plots ($\log_2$ expression level vs. $\log_2$ mean expression) (**D, E, F**). Proteins were considered as differentially expressed with a *p*-value < 0.05 and a fold change > 1.5 (students *t*-test). Only proteins with quantitative values in all biological replicates (n=3) of both compared groups were tested.

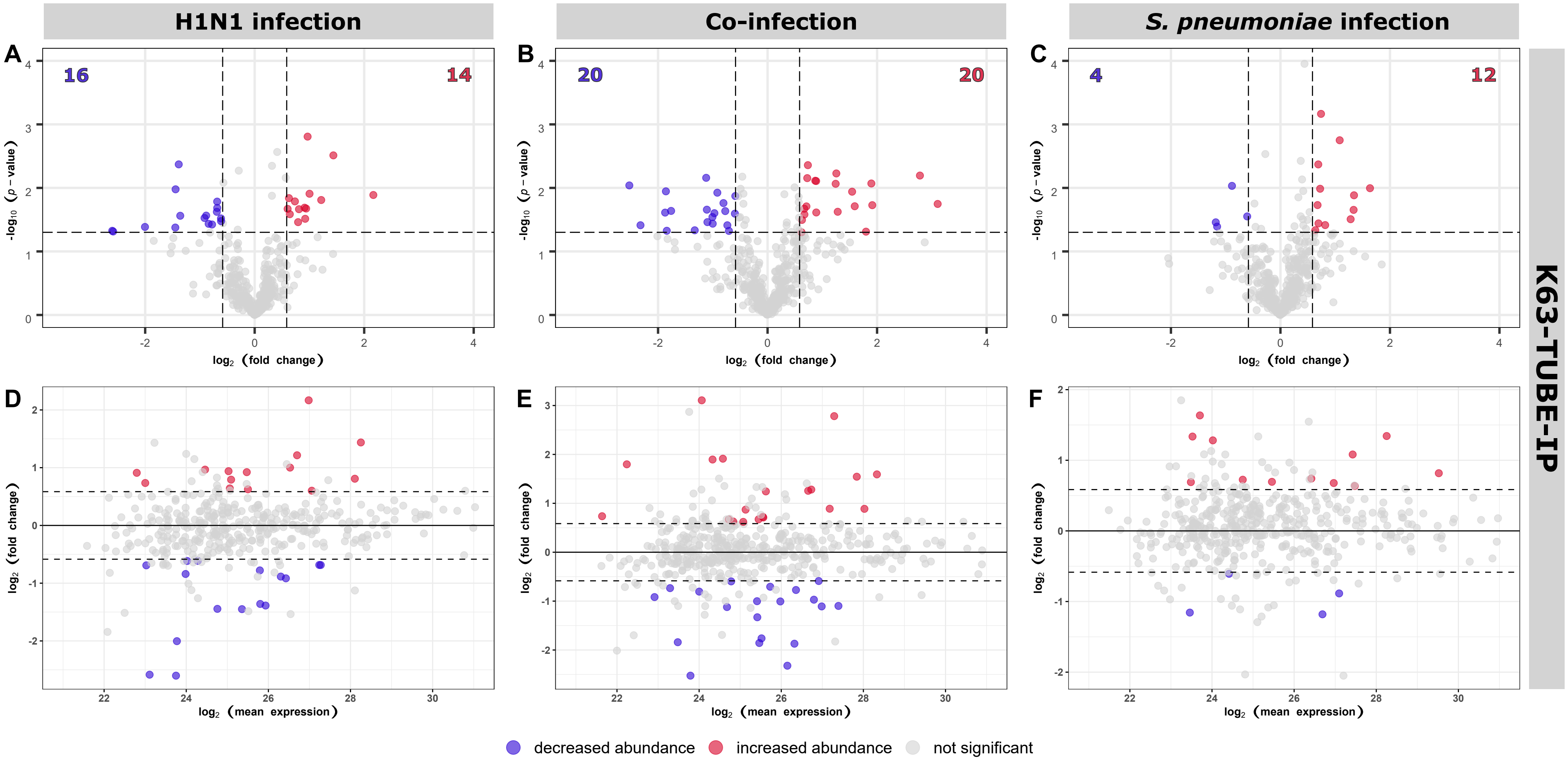

Figure S8: Results of the differential expression analysis comparing the uninfected control with the respective infection (**A** and **D** - H1N1 infection; **B** and **E** - co-infection; **C** and **F** - *S. pneumoniae* D39Δ*cps* infection) of K63 polyubiquitin enriched samples displayed as volcano plots (-$\log_{10}$ *p*-value vs. $\log_2$ expression level) (**A, B, C**) and as MA-plots ($\log_2$ expression level vs. $\log_2$ mean expression) (**D, E, F**). Proteins were considered as differentially expressed with a *p*-value < 0.05 and a fold change > 1.5 (students *t*-test). Only proteins with quantitative values in all biological replicates (n=3) of both compared groups were tested.

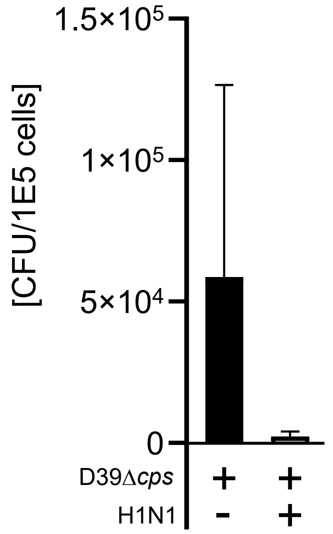

Figrure S9: Adherence of *Streptococcus pneumoniae* D39Δ*cps* to A549 epithelial cells alone and upon preceding viral infection for 24 h (A/Germany-BY/74/2009(H1N1) at a MOI of 5). Pneumococci were added to the A549 monolayer at a MOI of 15 and incubated for 6 h at 37 °C and 5% CO_2. After the period of infection cells were washed with phosphate buffered saline (PBS) and incubated with Tergitol for 10 minutes. Bacterial cell counts were obtained by counting colony forming units after plating serial dilutions on Columbia 5% sheep blood agar. Data are presented as the mean + SD [n=3] (no statistical difference was detected by applying a *t*-test). Preceding influenza A virus infection is commonly accepted to promote secondary infections and increase pneumococcal adherence to host cells. Therefore, we expected pneumococcal adherence to be at a similar or elevated level when comparing the co-infection to the single infection. Nevertheless, in the presented study we observed reduced pneumococcal adherence upon preceding viral infection. It cannot be concluded whether this is caused by the applied viral strain, the experimental conditions or a combination of multiple factors. Thus, we decided to use the 2009 pandemic H1N1 strain over a more common laboratory strain, like the PR8 strain. Still, the reduction of pneumococcal adherence to epithelial cells upon preceding viral infection is in contrast to common understanding. However, the effects on ubiquitination observed in the pneumococcal single infection were detected in the co-infection, as well. Together, the results of this study allow the conclusion that the co-infection, under the selected experimental conditions, shows additive, rather than synergistic effects. Further analyses are necessary to check if adherence of pneumococci to host cells is reduced upon preceding viral infection with the influenza strain used in this study.

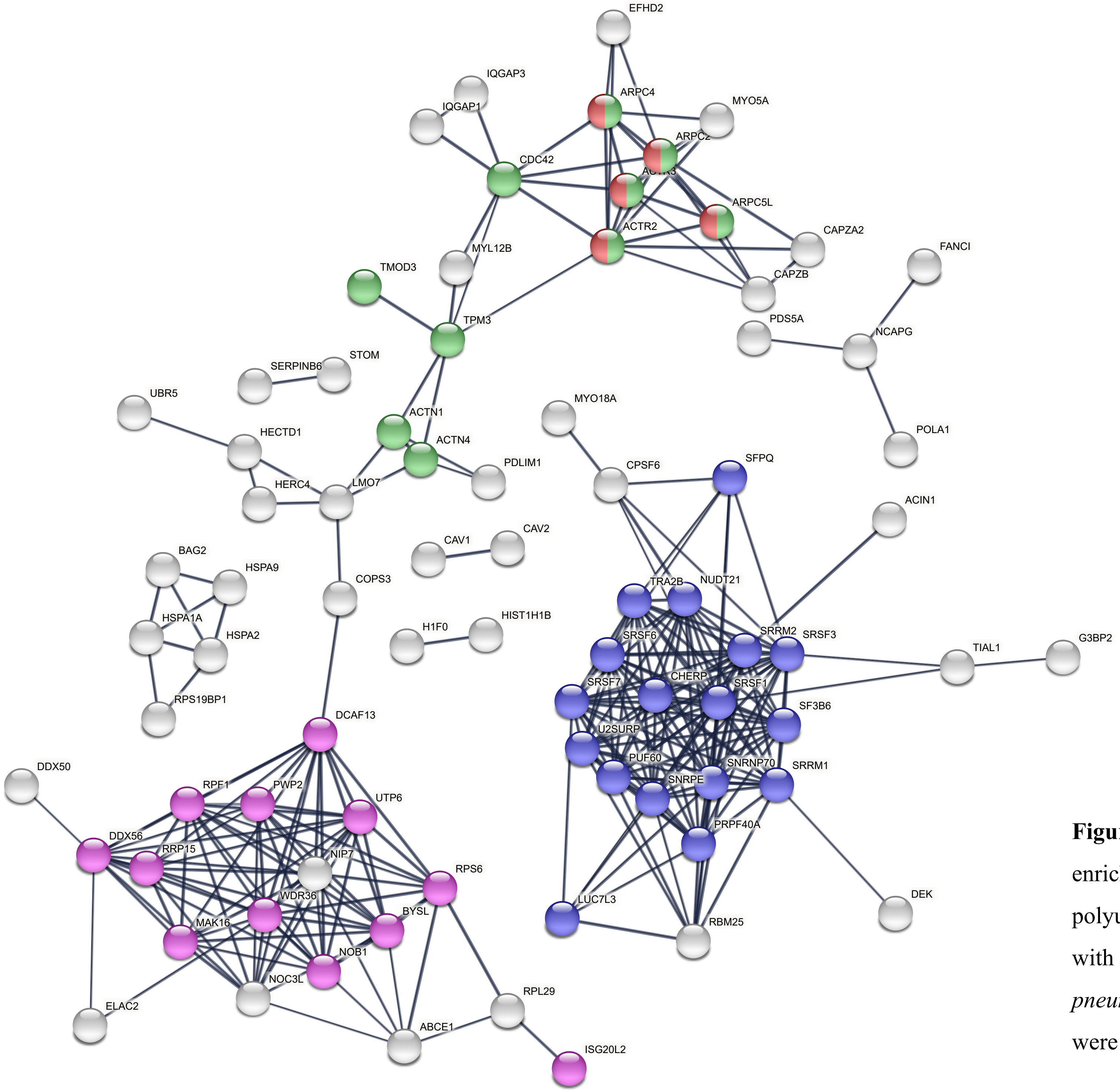

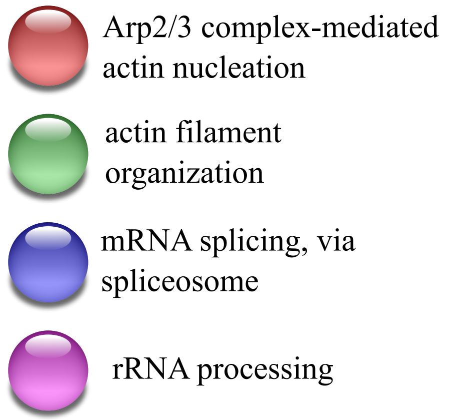

Figure S10: STRING network analysis and the enriched Reactome pathways from K48 polyubiquitinated proteins. Network from proteins with differential expression after *Streptococcus pneumoniae* D39Δ*cps* single infection. Experiments were performed in triplicates (n=3).

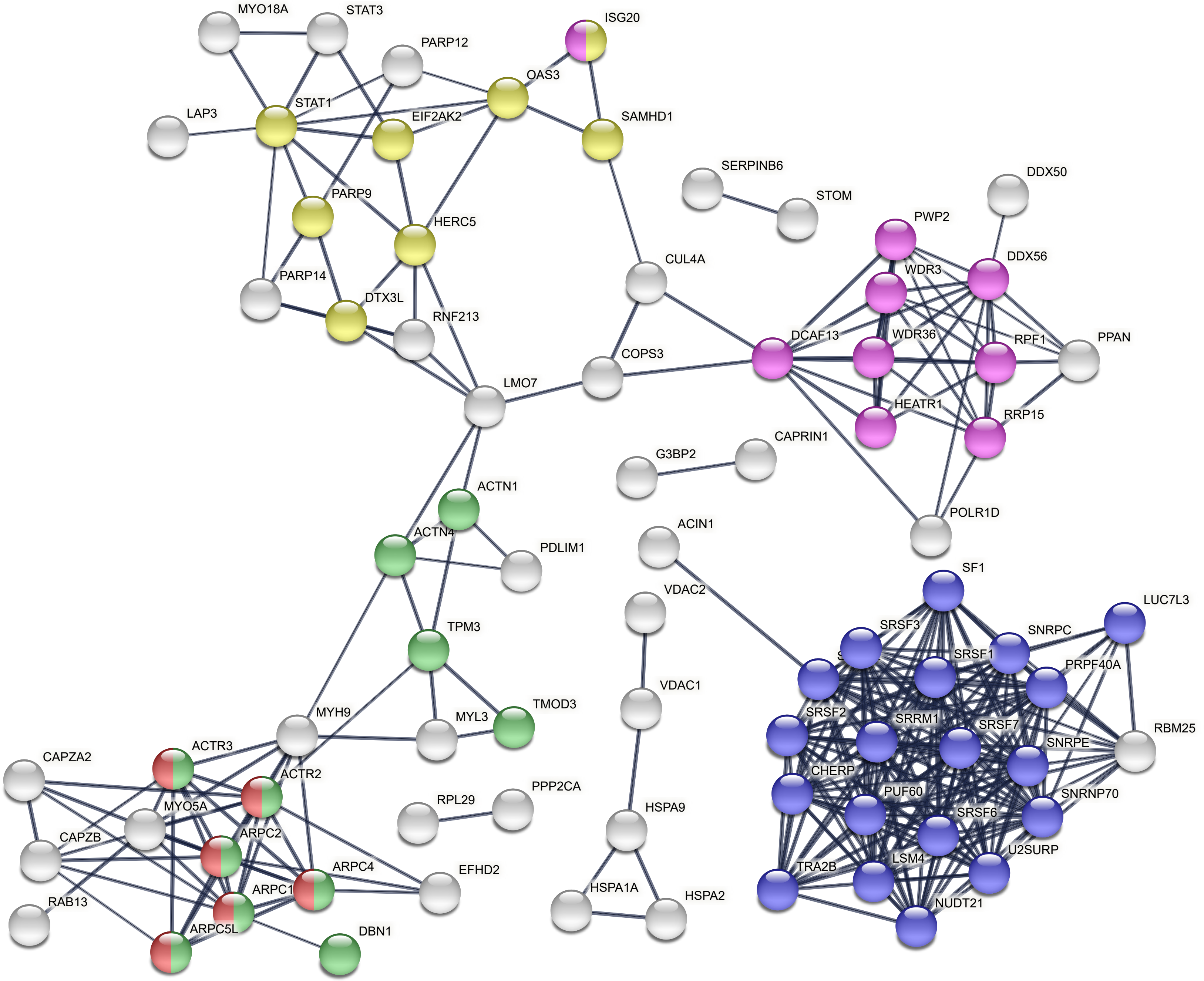

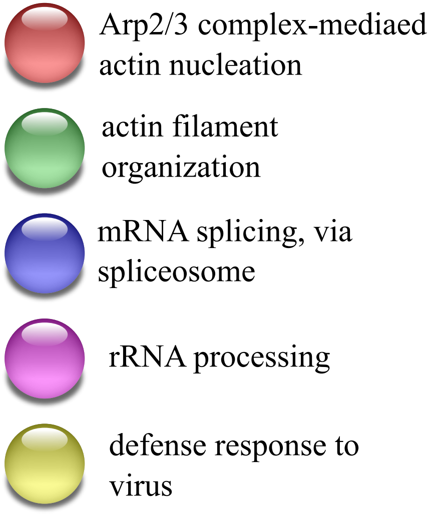

Figure S11: STRING network analysis and the enriched Reactome pathways from K48 polyubiquitinated proteins. Network from proteins with differential expression after co-infection with influenza A virus and *Streptococcus pneumoniae* D39Δ*cps*. Experiments were performed in triplicates (n=3).

Table S1 LC parameters

LC-Parameters	
Instrument	Easy-nLC 1000 (Thermo Fisher Scientific)
Analytical column	In house packed, 20 cm, 75 µm ID, 1.9 µm, 120 Å pore size; Dr. Maisch ReproSil-Pur 120 C18-AQ
Solvent system	Binary solvent system consisting of 0.1% acetic acid in *A. dest.* (solvent A) and 0.1% acetic acid in ACN (solvent B)
Flow rate	300 nl/min
Gradient	0 min 2% B<br>3 min 5% B<br>138 min 25% B<br>165 min 50% B<br>166 min 99% B<br>174 min 99% B<br>175 min 1% B<br>180 min 1% B
Method duration	180 min

Table S2 MS/MS parameters for data acquisition for proteome and K48 enriched samples

**MS-Parameters**	
Instrument	Q Exactive mass spectrometer (Thermo Fisher Scientific)
Operation mode	Data-dependent acquisition
Runtime	180 min
Lock mass correction	enabled
**Full MS-Parameters**	
MS scan resolution	140,000
AGC target	3e6
Maximum ion injection time for the MS scan	120 ms
Scan range	350 to 1650 m/z
Spectra data type	Profile
**MS2-Parameters**	
Resolution	17,500
MS/MS AGC target	1e5
Maximum ion injection time for the MS/MS scans	60 ms
Selection for MS/MS	15 most abundant isotope patterns with charge ≥2 from the survey scan
Isolation window	2 m/z
Fixed first mass	-
Dissociation mode	Higher energy collisional dissociation (HCD)
Normalized collision energy	27
Spectra data type	Centroid
Minimum AGC target	5e3
Intensity threshold	8.3e4
Dynamic exclusion	30 s
Charge exclusion	unassigned, 1, ≥7

Table S3 MS/MS parameters for data acquisition of K63 enriched samples

**MS-Parameters**	
Instrument	Q Exactive mass spectrometer (Thermo Fisher Scientific)
Operation mode	Data-dependent acquisition
Runtime	180 min
Lock mass correction	enabled
**Full MS-Parameters**	
MS scan resolution	70,000
AGC target	3e6
Maximum ion injection time for the MS scan	120 ms
Scan range	350 to 1650 m/z
Spectra data type	Profile
**MS2-Parameters**	
Resolution	17,500
MS/MS AGC target	1e5
Maximum ion injection time for the MS/MS scans	180 ms
Selection for MS/MS	10 most abundant isotope patterns with charge ≥2 from the survey scan
Isolation window	2 m/z
Fixed first mass	-
Dissociation mode	Higher energy collisional dissociation (HCD)
Normalized collision energy	27
Spectra data type	Centroid
Minimum AGC target	1e4
Intensity threshold	5.6e4
Dynamic exclusion	30 s
Charge exclusion	unassigned, 1, ≥7

Table S4 MaxQuant parameters for protein identification and quantification to analyze the proteome

**Parameter**	**Value**
Version	1.6.17.0
Include contaminants	TRUE
PSM FDR	0.01
PSM FDR Crosslink	0.01
Protein FDR	0.01
Site FDR	0.01
Use Normalized Ratios For Occupancy	TRUE
Min. peptide Length	7
Min. score for unmodified peptides	0
Min. score for modified peptides	40
Min. delta score for unmodified peptides	0
Min. delta score for modified peptides	6
Min. unique peptides	2
Min. razor peptides	2
Min. peptides	2
Use only unmodified peptides and	TRUE
Modifications included in protein quantification	Oxidation (M);Acetyl (Protein N-term)
Peptides used for protein quantification	Unique
Discard unmodified counterpart peptides	TRUE
Label min. ratio count	2
Use delta score	FALSE
iBAQ	TRUE
iBAQ log fit	TRUE
Match between runs	TRUE
Matching time window [min]	0.7
Match ion mobility window [indices]	0.05
Alignment time window [min]	10
Alignment ion mobility window [indices]	1
Find dependent peptides	FALSE
Fasta file	F:\Thomas\171215_Influenza_A__Germany_BY_74_2009_H1N1_reference_proteome_uniprot.fasta;F:\Thomas\190715_human_reference_uniprot_proteome_AUP000005640_reviewed.fasta;F:\Thomas\200922_S_pneumoniae_D39_UP000001452.fasta
Decoy mode	revert

Parameter	Value
Include contaminants	TRUE
Advanced ratios	TRUE
Second peptides	TRUE
Stabilize large LFQ ratios	TRUE
Separate LFQ in parameter groups	FALSE
Require MS/MS for LFQ comparisons	TRUE
Calculate peak properties	FALSE
Main search max. combinations	200
Advanced site intensities	TRUE
Write msScans table	FALSE
Write msmsScans table	TRUE
Write ms3Scans table	TRUE
Write allPeptides table	TRUE
Write mzRange table	TRUE
Write DIA fragments table	FALSE
Write pasefMsmsScans table	TRUE
Write accumulatedPasefMsmsScans table	TRUE
Max. peptide mass [Da]	4600
Min. peptide length for unspecific search	8
Max. peptide length for unspecific search	25
Razor protein FDR	TRUE
Disable MD5	FALSE
Max mods in site table	3
Match unidentified features	FALSE
Evaluate variant peptides separately	TRUE
Variation mode	None
MS/MS tol. (FTMS)	20 ppm
Top MS/MS peaks per Da interval. (FTMS)	12
Da interval. (FTMS)	100
MS/MS deisotoping (FTMS)	TRUE
MS/MS deisotoping tolerance (FTMS)	7
MS/MS deisotoping tolerance unit (FTMS)	ppm
MS/MS higher charges (FTMS)	TRUE
MS/MS water loss (FTMS)	TRUE
MS/MS ammonia loss (FTMS)	TRUE
MS/MS dependent losses (FTMS)	TRUE

Parameter	Value
MS/MS recalibration (FTMS)	FALSE
MS/MS tol. (ITMS)	0.5 Da
Top MS/MS peaks per Da interval. (ITMS)	8
Da interval. (ITMS)	100
MS/MS deisotoping (ITMS)	FALSE
MS/MS deisotoping tolerance (ITMS)	0.15
MS/MS deisotoping tolerance unit (ITMS)	Da
MS/MS higher charges (ITMS)	TRUE
MS/MS water loss (ITMS)	TRUE
MS/MS ammonia loss (ITMS)	TRUE
MS/MS dependent losses (ITMS)	TRUE
MS/MS recalibration (ITMS)	FALSE
MS/MS tol. (TOF)	40 ppm
Top MS/MS peaks per Da interval. (TOF)	10
Da interval. (TOF)	100
MS/MS deisotoping (TOF)	TRUE
MS/MS deisotoping tolerance (TOF)	0.01
MS/MS deisotoping tolerance unit (TOF)	Da
MS/MS higher charges (TOF)	TRUE
MS/MS water loss (TOF)	TRUE
MS/MS ammonia loss (TOF)	TRUE
MS/MS dependent losses (TOF)	TRUE
MS/MS recalibration (TOF)	FALSE
MS/MS tol. (Unknown)	20 ppm
Top MS/MS peaks per Da interval. (Unknown)	12
Da interval. (Unknown)	100
MS/MS deisotoping (Unknown)	TRUE
MS/MS deisotoping tolerance (Unknown)	7
MS/MS deisotoping tolerance unit (Unknown)	ppm
MS/MS higher charges (Unknown)	TRUE
MS/MS water loss (Unknown)	TRUE
MS/MS ammonia loss (Unknown)	TRUE
MS/MS dependent losses (Unknown)	TRUE
MS/MS recalibration (Unknown)	FALSE

Parameter	Value
Site tables	Oxidation (M)Sites.txt

Table S5 MaxQuant parameters for protein identification and quantification to analyze K48 and K63 enriched samples

**Parameter**	**Value**
Version	1.6.17.0
Include contaminants	TRUE
PSM FDR	0.01
PSM FDR Crosslink	0.01
Protein FDR	0.01
Site FDR	0.01
Use Normalized Ratios For Occupancy	TRUE
Min. peptide Length	7
Min. score for unmodified peptides	0
Min. score for modified peptides	40
Min. delta score for unmodified peptides	0
Min. delta score for modified peptides	6
Min. unique peptides	2
Min. razor peptides	2
Min. peptides	2
Use only unmodified peptides and	TRUE
Modifications included in protein quantification	Oxidation (M);Acetyl (Protein N-term);GlyGly (K)
Peptides used for protein quantification	Unique
Discard unmodified counterpart peptides	TRUE
Label min. ratio count	2
Use delta score	FALSE
iBAQ	TRUE
iBAQ log fit	TRUE
Match between runs	TRUE
Matching time window [min]	0.7
Match ion mobility window [indices]	0.05
Alignment time window [min]	10
Alignment ion mobility window [indices]	1
Find dependent peptides	FALSE
Fasta file	F:\Thomas\171215_Influenza_A__Germany_BY_74_2009_H1N1_reference_proteome_uniprot.fasta;F:\Thomas\190715_human_reference_uniprot_proteome_AUP000005640_reviewed.fasta;F:\Thomas\200922_S_pneumoniae_D39_UP000001452.fasta

Parameter	Value
First search fasta file	F:\Thomas\190715_human_reference_uniprot_proteome_AUP000005640_reviewed.fasta
Decoy mode	revert
Include contaminants	TRUE
Advanced ratios	TRUE
Second peptides	TRUE
Stabilize large LFQ ratios	TRUE
Separate LFQ in parameter groups	FALSE
Require MS/MS for LFQ comparisons	TRUE
Calculate peak properties	FALSE
Main search max. combinations	200
Advanced site intensities	TRUE
Write msScans table	FALSE
Write msmsScans table	TRUE
Write ms3Scans table	TRUE
Write allPeptides table	TRUE
Write mzRange table	TRUE
Write DIA fragments table	FALSE
Write pasefMsmsScans table	TRUE
Write accumulatedPasefMsmsScans table	TRUE
Max. peptide mass [Da]	4600
Min. peptide length for unspecific search	8
Max. peptide length for unspecific search	25
Razor protein FDR	TRUE
Disable MD5	FALSE
Max mods in site table	3
Match unidentified features	FALSE
Evaluate variant peptides separately	TRUE
Variation mode	None
MS/MS tol. (FTMS)	20 ppm
Top MS/MS peaks per Da interval. (FTMS)	12
Da interval. (FTMS)	100
MS/MS deisotoping (FTMS)	TRUE
MS/MS deisotoping tolerance (FTMS)	7
MS/MS deisotoping tolerance unit (FTMS)	ppm
MS/MS higher charges (FTMS)	TRUE

Parameter	Value
MS/MS water loss (FTMS)	TRUE
MS/MS ammonia loss (FTMS)	TRUE
MS/MS dependent losses (FTMS)	TRUE
MS/MS recalibration (FTMS)	FALSE
MS/MS tol. (ITMS)	0.5 Da
Top MS/MS peaks per Da interval. (ITMS)	8
Da interval. (ITMS)	100
MS/MS deisotoping (ITMS)	FALSE
MS/MS deisotoping tolerance (ITMS)	0.15
MS/MS deisotoping tolerance unit (ITMS)	Da
MS/MS higher charges (ITMS)	TRUE
MS/MS water loss (ITMS)	TRUE
MS/MS ammonia loss (ITMS)	TRUE
MS/MS dependent losses (ITMS)	TRUE
MS/MS recalibration (ITMS)	FALSE
MS/MS tol. (TOF)	40 ppm
Top MS/MS peaks per Da interval. (TOF)	10
Da interval. (TOF)	100
MS/MS deisotoping (TOF)	TRUE
MS/MS deisotoping tolerance (TOF)	0.01
MS/MS deisotoping tolerance unit (TOF)	Da
MS/MS higher charges (TOF)	TRUE
MS/MS water loss (TOF)	TRUE
MS/MS ammonia loss (TOF)	TRUE
MS/MS dependent losses (TOF)	TRUE
MS/MS recalibration (TOF)	FALSE
MS/MS tol. (Unknown)	20 ppm
Top MS/MS peaks per Da interval. (Unknown)	12
Da interval. (Unknown)	100
MS/MS deisotoping (Unknown)	TRUE
MS/MS deisotoping tolerance (Unknown)	7
MS/MS deisotoping tolerance unit (Unknown)	ppm
MS/MS higher charges (Unknown)	TRUE
MS/MS water loss (Unknown)	TRUE

**Parameter**	**Value**
MS/MS ammonia loss (Unknown)	TRUE
MS/MS dependent losses (Unknown)	TRUE
MS/MS recalibration (Unknown)	FALSE
Site tables	GlyGly (K)Sites.txt;Oxidation (M)Sites.txt
